# Marginal Ulcer and Dumping Syndrome in Patients after Duodenal Switch: A Multi-Centered Study

**DOI:** 10.3390/jcm12175600

**Published:** 2023-08-28

**Authors:** Marita Salame, Andre F. Teixeira, Romulo Lind, Gilberto Ungson, Muhammad Ghanem, Kamal Abi Mosleh, Muhammad A. Jawad, Barham K. Abu Dayyeh, Michael L. Kendrick, Omar M. Ghanem

**Affiliations:** 1 Department of Surgery, Mayo Clinic, Rochester, MN 55905, USA; 2 Department of Surgery, Orlando Health, Orlando, FL 32806, USA; 3 Department of Surgery, Cima Hospital, Hermosillo 83280, Mexico; 4 Division of Gastroenterology and Hepatology, Mayo Clinic, Rochester, MN 55905, USA

**Keywords:** marginal ulcer, dumping syndrome, duodenal switch, bariatric surgery

## Abstract

Background: The current design of biliopancreatic diversion with duodenal switch (BPD/DS) and single anastomosis duodenal–ileal bypass with sleeve (SADI-S) emphasizes the importance of the pylorus’ preservation to reduce the incidence of marginal ulcer (MU) and dumping. However, no institutional studies have yet reported data on their prevalence. We aimed to assess the incidence of MU and dumping after duodenal switch (DS) and identify the associative factors. Methods: A multi-center review of patients who underwent BPD/DS or SADI-S between 2008 and 2022. Baseline demographics, symptoms, and management of both complications were collected. Fisher’s exact test was used for categorical variables and the independent *t*-test for continuous variables. Results: A total of 919 patients were included (74.6% female; age 42.5 years; BMI 54.6 kg/m^2^) with mean follow-up of 31.5 months. Eight patients (0.9%) developed MU and seven (0.8%) had dumping. Patients who developed MU were more likely to be using non-steroidal anti-inflammatory drugs (NSAID) (*p* = 0.006) and have a longer operation time (*p* = 0.047). Primary versus revisional surgery, and BDP/DS versus SADI-S were not associated with MU or dumping. Conclusions: The incidences of MU and dumping after DS were low. NSAID use and a longer operation time were associated with an increased risk of MU, whereas dumping was attributed to poor dietary habits.

## 1. Introduction

Obesity constitutes a major health risk and is the root of many diseases, leading to increased mortality and morbidity worldwide [1]. Metabolic and bariatric surgery (MBS) is currently considered the most effective long-term treatment for obesity, with duodenal switch (DS) leading the way in terms of sustained weight loss and resolution of obesity-related comorbidities [2]. However, its adoption has been slow, partially because of its complexity, lack of training, and persistent concerns about long-term complications [3]. 

The current design of the biliopancreatic diversion with duodenal switch (BPD/DS) was created by Hess et al. as a way to address the limitations of the initial description of biliopancreatic diversion surgery by Scopinaro in 1979, which had a high risk of nutritional deficiencies and postoperative complications such as marginal ulcer (MU) and dumping syndrome [4]. The single anastomosis duodenal–ileal bypass with sleeve (SADI-S) was later introduced in 2007 as a simplified version of BPD/DS while maintaining its effectiveness and has recently been endorsed by the American Society for Metabolic and Bariatric Surgery as a safe MBS [5,6]. Despite the several refinements made to the DS procedure over the years, preserving the pyloric sphincter has always been a priority in order to lower the rates of gastric emptying and minimize postoperative complications [7]. Nevertheless, patients undergoing DS may still be at risk of developing complications, such as MU and dumping syndrome. 

Both MU and dumping syndrome have been extensively studied after Roux-en-Y gastric bypass. In this patient population, MU occurs at or in proximity to the gastrojejunal anastomosis and can lead to a range of symptoms such as abdominal pain, nausea, vomiting, and bleeding, with some patients being asymptomatic. Its pathophysiology is multifactorial, and several risk factors such as gastric acid hypersecretion, diabetes mellitus, *Helicobacter pylori* infection, tobacco, non-steroidal anti-inflammatory drugs (NSAID), and corticosteroids use have been reported in many previous studies [8,9,10,11,12]. In severe cases, MU may lead to perforation, necessitating urgent treatment [12]. Dumping syndrome, on the other hand, is a collection of symptoms that occur due to rapid emptying of food into the small intestine, causing symptoms such as abdominal cramps, nausea, diarrhea, and weakness [13]. These complications can significantly impact the quality of life of patients, and can also result in the need for readmission, reoperation, and reintervention. 

While both complications may also occur after DS, there is a paucity of literature that investigates the prevalence and risk factors of these complications in a large cohort of patients undergoing DS. Therefore, we aimed to assess the incidence of MU and dumping in patients who underwent BPD/DS and SADI-S and identify the associative factors.

## 2. Materials and Methods

### 2.1. Patient Selection

We performed a retrospective chart review of all patients who underwent BPD/DS and SADI-S at 2 different tertiary referral centers for bariatric surgeries from January 2008 to June 2022. We included patients who were ≥18 years of age and who underwent BPD/DS or SADI-S as a primary or revisional procedure. All patients underwent a thorough medical preoperative assessment. Patients were advised to eliminate the use of alcohol and tobacco prior to surgery. Weight related comorbidities were diagnosed by the treating physician in the preoperative evaluation. After obtaining approval from our institutional review board, we collected data on patients’ baseline characteristics, demographics, comorbidities, perioperative outcomes, and complications. The patient baseline was established using the most recent available data before the bariatric procedure. To ensure patient confidentiality and data protection, all patient information was de-identified throughout the study. 

### 2.2. Operative Technique

Initially, both institutions implemented BPD/DS procedures, subsequently introducing SADI-S into their evolving protocols. These surgical interventions were performed either through laparoscopic or robotic techniques. All procedures were performed using a standardized technique. The sleeve was tailored around a 50 French bougie. The duodenal dissection was performed until the gastroduodenal artery was encountered, allowing for at least 2–3 cm of a transected duodenal cuff for the duodeno-ileostomy. The duodeno-ileostomy anastomosis was created using a two-layer handsewn closure in all cases. In the SADI-S procedure, a common limb length of 300 cm was used. On the other hand, in the BPD/DS procedure, the common limb length ranged between 100 and 150 cm, while the Roux limb length varied between 125 and 150 cm. 

### 2.3. Endpoints

The primary outcome of this study is to determine the incidence of MU and dumping syndrome in patients who underwent BPD/DS and SADI-S. MU is defined as ulceration of the duodenoileal anastomosis, and dumping syndrome is defined as the presence of symptoms consistent with early or late dumping. Secondary outcomes include identifying potential risk factors associated with these postoperative complications. 

### 2.4. Diagnosis

The diagnosis of MU was based on clinical findings and was confirmed endoscopically by an expert gastroenterologist or bariatric surgeon. The diagnosis of dumping syndrome was established by reviewing clinical notes provided by medical experts, including endocrinologists and bariatricians, which relied on symptoms and laboratory findings. During this process, common differential diagnoses, such as small intestinal bacterial overgrowth and malabsorptive diarrhea, were excluded.

### 2.5. Statistical Analysis

Descriptive statistics were used to summarize patient demographics and surgical details. Data were collected from electronic health records and analyzed using IBM SPSS statistics version 27. All variables were normally distributed and hence parametric tests were used accordingly. Categorical variables were analyzed using Fisher’s exact test as appropriate and summarized as frequencies (n) and percentages (%). Continuous variables were analyzed using the independent samples *t*-test and were summarized as mean ± standard deviation. Statistical significance was set at *p*-value < 0.05.

## 3. Results

A total of 919 consecutive patients were included in the study, with a mean follow-up period of 31.5 ± 20.8 months. The majority of the patients were female (74.6%), with a mean age of 42.5 ± 9.9 years and mean body mass index (BMI) of 54.6 ± 9.7 kg/m^2^. Most patients were White (72.3%) or African American/Black (18.5%). Of these patients, 774 (84.2%) underwent BPD/DS, and 145 (15.8%) had SADI-S, with a mean operation (OR) time of 168 ± 73 min. Over 75% of patients had no history of gastroesophageal reflux (GERD), and 92.4% were non-smokers. Sleep apnea was present in less than 50% of patients, and 20.4% reported NSAID use. The baseline patient demographics are reviewed in Table 1.

**Figure 1:**
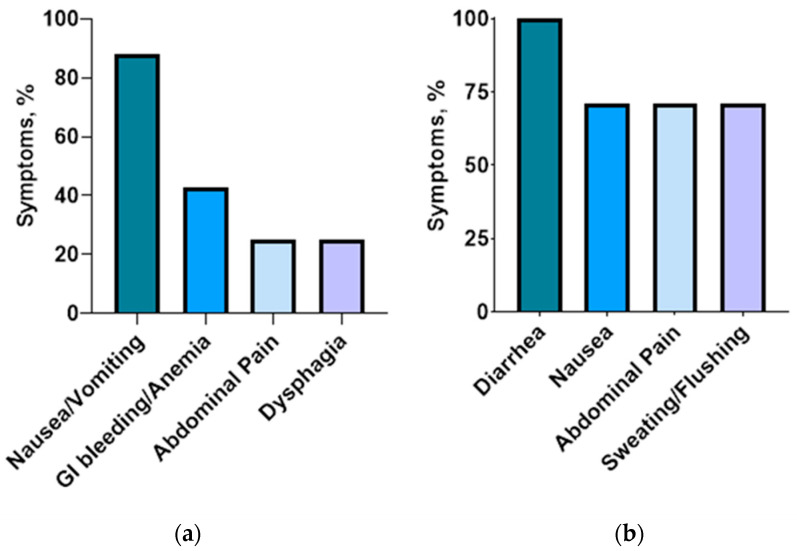
Symptoms of marginal ulcer (**a**) and dumping syndrome (**b**).

Among the entire cohort, the incidence of MU was 0.9% (8 out of 919). All patients who developed MU had undergone BPD/DS, with a mean OR time of 227 ± 69 min. Of these patients, six (75%) had hypertension, four (50%) had diabetes, and two had GERD. None were current smokers, but 80% used NSAIDs. Five patients (62.5%) developed MU within 6 months after surgery. Symptoms were reported in all cases, with nausea/vomiting being the most common (87.5%), followed by gastrointestinal bleeding and anemia (42.9%). Other presenting symptoms can be found in Figure 1a. Only one patient (12.5%) required surgery for treatment, while the remaining 87.5% were managed medically with proton pump inhibitors, sucralfate, and lifestyle modifications. All patients had healed completely with treatment, with no recurrence. Patients who developed MU were significantly more likely to be using NSAIDs (*p* = 0.006) and have longer OR time (*p* = 0.047). There was no significant association between MU and GERD (*p* = 1.0), diabetes (*p* = 0.24), smoking (*p* = 1.0), or sleep apnea (*p* = 0.16). Primary versus revisional surgery (*p* = 0.63) and BDP/DS versus SADI-S (*p* = 0.62) had no statistically significant association with the development of MU.

**Figure 2:**
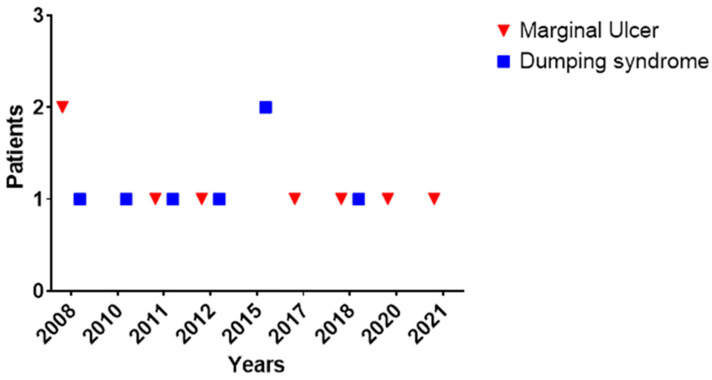
Timeline of marginal ulcer and dumping diagnoses between 2008 and 2022.

On the other hand, seven patients (0.8%) experienced dumping syndrome after BPD/DS. More than half (57.1%) of the patients developed dumping within 6 months following the surgery. Hypoglycemia was reported in 85.7% of patients (n = 6), with mean glycemia levels of 55.7 ± 28.6 mg/L. Fifty percent of patients had early dumping, while the remaining fifty percent experienced late dumping. Dumping was reported by five patients after consuming foods high in fat or sugar, while two experienced dumping when they consumed liquids with meals. Additionally, two patients reported dumping after overeating or consuming their meals too quickly. All patients had coexistent sleep apnea, with 42.9% having diabetes, 57.1% having hypertension, and 14.3% being current smokers. Diarrhea was experienced by all patients, while 71.4% of them also reported nausea and sweating/ flushing (Figure 1b). One patient underwent a provocative test—the mixed-meal tolerance test—while the rest were clinically diagnosed. All patients were successfully treated through lifestyle modifications and dietary changes, without the need for medical treatment and revisional surgery. There was no statistically significant association found between the occurrence of dumping syndrome and either primary versus revisional surgery (*p* = 0.33) or BPD/DS versus SADI-S (*p* = 0.60). The occurrence of dumping syndrome and MU was evenly distributed across all the years (Figure 2).

## 4. Discussion

To the extent of our knowledge, this is the first multi-centered study assessing the prevalence of both MU and dumping syndrome after DS in the United States. Our findings show low incidence of MU and dumping syndrome following DS. While longer OR time and NSAID use were associated with higher risk of MU, dumping, in our cohort, was attributed with poor dietary habits. These findings could help to guide patient counseling and surgical decision making in the context of DS.

In our study, we reported a low incidence of MU, which aligns with the limited available research reporting MU rates ranging from 0.2% to 1.9% in patients following DS [4,14,15,16,17]. This low occurrence of MU in DS could be attributed to the preservation of the pyloric sphincter, which plays a crucial role in buffering and neutralizing the substantial amount of gastric acid that passes into the small bowel after the procedure [18]. In fact, the alkaline mucus produced by the Brunner’s glands in the duodenum plays a role in protecting the ileal mucosa from the high acidity as well as the reduced parietal cell mass from the sleeve gastrectomy [7]. An experimental study conducted in 1987 by DeMeester on animal models demonstrated a decreased incidence of ulceration after preserving the pylorus [19] and observational studies have confirmed these findings [4,15]. 

In the general population, the occurrence of peptic ulcers is estimated to be between 5 and 10% throughout a person’s lifetime, with an annual incidence of 0.1–0.3% [20]. This rate was comparable to the rates reported in our DS cohort, considering that they were followed up for a mean period of 32 months, therefore, suggesting that the rates do not significantly increase in patients post DS. When compared to other bariatric surgery procedures such as Roux-en-Y gastric bypass (RYGB) and one anastomosis gastric bypass (OAGB), the incidence of MU following DS is relatively low. In fact, MU is a well-recognized complication following RYGB, with an incidence rate of up to 25% in the literature [11]. Moreover, a study of De la Cruz compared the incidence of anastomotic ulcers in patients that underwent SADI-S and OAGB, and found that no ulcer cases developed in the DS group, compared to an incidence of 2.4% in the OAGB group [21]. The absence of any MU cases in the SADI-S portion of our DS cohort highlights the effectiveness of this procedure at minimizing the risk and aligns with several studies [22,23]. This stresses the role of pylorus preservation, since stomach transection is performed in the preantral region followed by a gastrojejunal anastomosis in the OAGB.

Several potential risk factors were associated with MU after bariatric surgery. Our study found a significant association between the use of NSAID and the development of MU, a finding consistent with several previous studies in the gastric bypass group [8,9,10,12]. A potential explanation is the mucosal disruption caused by the decrease in prostaglandins and blood flow, subsequently leading to reduced bicarbonate and mucus secretion following NSAID use [24]. Therefore, it might be safer to minimize NSAID use after DS. Moreover, we found an association between longer OR time and MU formation. Prolonged operative durations, especially when conducted by the same surgical team, often indicate a higher level of surgical complexity. A reasonable explanation might be pyloric damage and insufficient length of the duodenal cuff in the more difficult cases requiring more OR time. Additionally, among the frequent contributing factors for longer OR time in patients undergoing duodenal switch is the challenge in transposing the ileum to the upper abdomen to reach the duodenum. This difficulty can lead to anastomotic tension, which increases the risk of complications such as leaks, stenosis, and ischemia, consequently increasing the likelihood of MU formation [12]. 

The incidence of dumping syndrome was found to be low following DS in our cohort. Originally, Hess and Marceau designed the BPD/DS procedure to alleviate postgastrectomy symptoms that were associated with the distal gastrectomy component of the original biliopancreatic diversion described in 1979, reporting no instances of dumping syndrome in their studies [4,7]. In addition, the anterior vagal branches, which provide the main innervation to the pylorus, are severed during the mobilization of the pyloric region, leading to alterations in the gastric emptying and antral function after DS [25]. Hedberg et al. conducted a study that confirmed a faster rate of gastric emptying in individuals who underwent BPD/DS compared to those who did not have surgery, despite the preservation of the pylorus. BPD/DS patients experience the benefits of a hormonal mechanism characterized by consistently elevated levels of peptide YY in the fasting state with levels rapidly rising in response to meals. This hormonal interplay further influences the dynamics of gastric motility and can contribute to the development of dumping syndrome [26]. Moreover, this could be attributed to the partial disruption of finely tuned pyloric function due to the dissection in the surrounding area, despite the anatomical preservation of the pylorus [26]. While there have been no published studies on dumping syndrome following DS, there are some available data after other bariatric procedures, with reported rates between 15–70%. The incidence of dumping depends on the type of gastrectomy performed, with a higher occurrence in patients who undergo total gastrectomy compared to proximal gastrectomy [27,28].

Dumping in our cohort was attributed to poor dietary choices and disregard for the recommended meal size and frequency, as well as drinking liquids during meals. These findings align with a recent study conducted by Alsulami et al., which showed a significant association between dietary habits and the occurrence of dumping. It was revealed that individuals who consumed more than one large meal had the highest prevalence of dumping, and those who drank liquids during meals had a significantly higher prevalence compared to those who drank liquids between meals [29]. Thus, it is recommended to focus on the reduction of simple carbohydrates, with a preference for high protein foods and consuming small meals without drinking liquids.

The findings of the study provide valuable insights into the potential complications associated with DS, particularly with BPD/DS. The presence of MU and dumping was observed at various times and distributed throughout all years, indicating that its incidence was not attributed to the learning curve or surgical expertise. However, there are some limitations that need to be acknowledged. Firstly, the study is observational in nature, which means that causality cannot be established. Secondly, the retrospective nature of the study does not allow for standardized patient diagnosis, management, and follow-up of patients. Thirdly, the follow-up period of 31.5 months may not be sufficient to capture long-term complications, which may emerge later in the postoperative period. As MU may be asymptomatic and only identifiable through routine endoscopy, its actual occurrence after DS might be higher, necessitating a systematic routine follow-up. Finally, due to missing information, we were unable to examine other potential risk factors for MU and dumping syndrome, such as dietary habits, alcohol use, and *Helicobacter pylori*, which could have possibly influenced the findings. 

## 5. Conclusions

The incidences of MU and dumping syndrome following BPD/DS and SADI-S were low. Patient selection, preoperative counseling, and postoperative monitoring, especially during the first six postoperative months, are important to ensure optimal outcomes. Avoiding NSAID use may be crucial to reduce the risk of MU, whereas following dietary recommendations is essential in preventing dumping syndrome.

## Figures and Tables

**Table 1:** Patient demographics and postoperative complications.

Demographic Information	All Patients
*N*	919
Age, years (SD)	42.5 (9.9)
Sex, Female (%)	686 (74.6)
Race, White (%)	664 (72.3)
Baseline Comorbidities and Risk Factors	
Weight, kg (SD)	155.7 (32.4)
BMI, kg/m^2^ (SD)	54.6 (9.7)
Operation time, min (SD)	168.9 (72.8)
Independent functional status (%)	916 (99.7)
GERD (%)	227 (24.7)
Hypertension (%)	467 (50.8)
Hyperlipidemia (%)	243 (26.4)
Sleep apnea (%)	433 (47.1)
T2DM (%)	267 (29.0)
Alcohol use (%)	25 (14.9)
NSAID use (%)	34 (20.4)
Current smoker (%)	70 (7.6)
Postoperative complications	
Marginal ulcer (%)	8 (0.9)
Dumping syndrome (%)	7 (0.8)

Abbreviations: BMI, body mass index; GERD, gastroesophageal reflux disease; T2DM, type 2 diabetes mellitus; NSAID, non-steroidal anti-inflammatory drugs. Data are presented as mean and standard deviation for continuous variables, and as frequency and percentage for categorical variables.

## Data Availability

The data presented in this study are available on request from the corresponding author. The data are not publicly available due to confidentiality.
